# Relationship of idiopathic femoral head necrosis with blood lipid metabolism and coagulation function: A propensity score-based analysis

**DOI:** 10.3389/fsurg.2022.938565

**Published:** 2023-01-06

**Authors:** Xiaolong Yu, Shengtao Zhang, Bin Zhang, Min Dai

**Affiliations:** Department of Orthopedics, Artificial Joints Engineering and Technology Research Center, The First Affiliated Hospital of Nanchang University, Nanchang, China

**Keywords:** femur head necrosis, lipid metabolism, coagulation function, propensity score matching, idiopathic necrosis of the femoral head

## Abstract

**Background:**

Nontraumatic osteonecrosis of the femoral head (ONFH) can be corticosteroid-induced, alcohol-induced, and idiopathic ONFH (IONFH). Although corticosteroid- and alcohol-induced ONFH has been investigated extensively regarding its relationship with blood lipids and coagulation factor levels. However, the effect of blood lipid metabolism and coagulation function on IONFH has rarely been studied. Therefore, this study aimed to analyse the relationship of IONFH with blood lipid and coagulation indicators.

**Methods:**

Total 680 patients diagnosed with IONFH in our institution during January 2011–June 2019 who met the inclusion criteria composed the case group; 613 healthy persons who underwent physical examination at our institution during the same period composed the control group. Propensity scores were used for baseline feature matching, and two matching groups each with 450 patients were established. After the matching, blood lipid and coagulation factor levels of both groups were comparatively analysed.

**Results:**

The case group showed significantly higher total cholesterol (TC), triglyceride (TG), low-density lipoprotein (LDL) levels, low-density/high-density lipoprotein (LDL/HDL) ratio, and apolipoprotein B (Apo-B) levels than the control group (*p* < 0.05). Conversely, the HDL and apolipoprotein A (Apo-AI) levels in the case group were significantly lower than those in the control group (*p* < 0.05). Regarding coagulation indicators, the activated partial thromboplastin time and prothrombin time were lower in the case group than in the control group; however, the differences were insignificant (*p* > 0.05). Furthermore, fibrinogen (FIB) levels and thrombin time (TT) in the case group were higher than those in the control group. There were significant differences between the two groups only in terms of FIB levels (*p* < 0.05), while TT was not significantly different (*p* > 0.05).

**Conclusions:**

IONFH has strong associations with blood lipid metabolism and coagulation function, which provide an avenue for exploring the mechanism of IONFH.

## Background

Osteonecrosis of the femoral head (ONFH) leads to the death of osteocytes and other bone marrow components due to interruption of blood supply to the femoral head that induces structural changes and collapse of the subchondral bone internally, which manifests as joint pain and dysfunction (1–3). Epidemiological data show that in the year 2000, the incidence of ONFH in the general population of the United States ranged from 300,000 to 600,000, with approximately 10,000 to 20,000 stable new cases each year (4, 5). Another study reported that the total number of patients with ONFH among the general population aged ≥15 years in China was about 8.12 million (6). Femoral head ischaemia can be divided into two categories: traumatic and nontraumatic ONFH. The aetiology of traumatic ONFH is direct vascular injury, blocking the blood flow to the femoral head, caused by a femoral neck fracture or dislocation of the hip joint (7). Currently, the aetiology of nontraumatic ONFH is not completely clear, which makes early diagnosis and treatment difficult.

Nontraumatic ONFH can be divided into three main aetiologic associations: corticosteroid-induced, alcohol-induced, and idiopathic ONFH (IONFH) (8). Corticosteroid use and alcohol intake are two of the most common causes of nontraumatic ONFH (9), accounting for about 40% of nontraumatic cases (10). Therefore, patients with these two factors can consciously take preventive measures and be examined timely for early diagnosis. However, some studies have shown that patients without corticosteroid- or alcohol-induced ONFH are regarded as patients with IONFH due to the unknown aetiology, such as spondylolisthesis of femoral head epiphysis, systemic lupus erythematosus, HIV infection, and hyperlipidaemia (8, 11, 12), indicating that IONFH is also an important cause of nontraumatic ONFH.

Corticosteroid- and alcohol-induced ONFH related to blood lipid metabolism and coagulation function has been investigated thoroughly (13–16). However, only a few reports probed on how lipid metabolism and coagulation function associate with IONFH. The aetiology of nontraumatic ONFH is mainly based on abnormal lipid metabolism and coagulation function (17, 18). Disorders of lipid metabolism may cause ischemia by increasing intraosseous pressure and decreasing blood flow ultimately leading to nontraumatic ONFH. In addition, insufficient fibrinolysis and thrombotic tendency also seem to play an essential role in nontraumatic ONFH. Given that IONFH is categorized as nontraumatic ONFH, it is necessary to elucidate the relationship between IONFH and lipid metabolism and coagulation function.

In this retrospective study, we hypothesised that most patients with IONFH had strong associations with blood lipid and coagulation factor levels. Therefore, the purpose of this study was to investigate the relationship between IONFH and endogenous lipid metabolism as well as coagulation.

## Methods

### Patient selection

**Figure 1:**
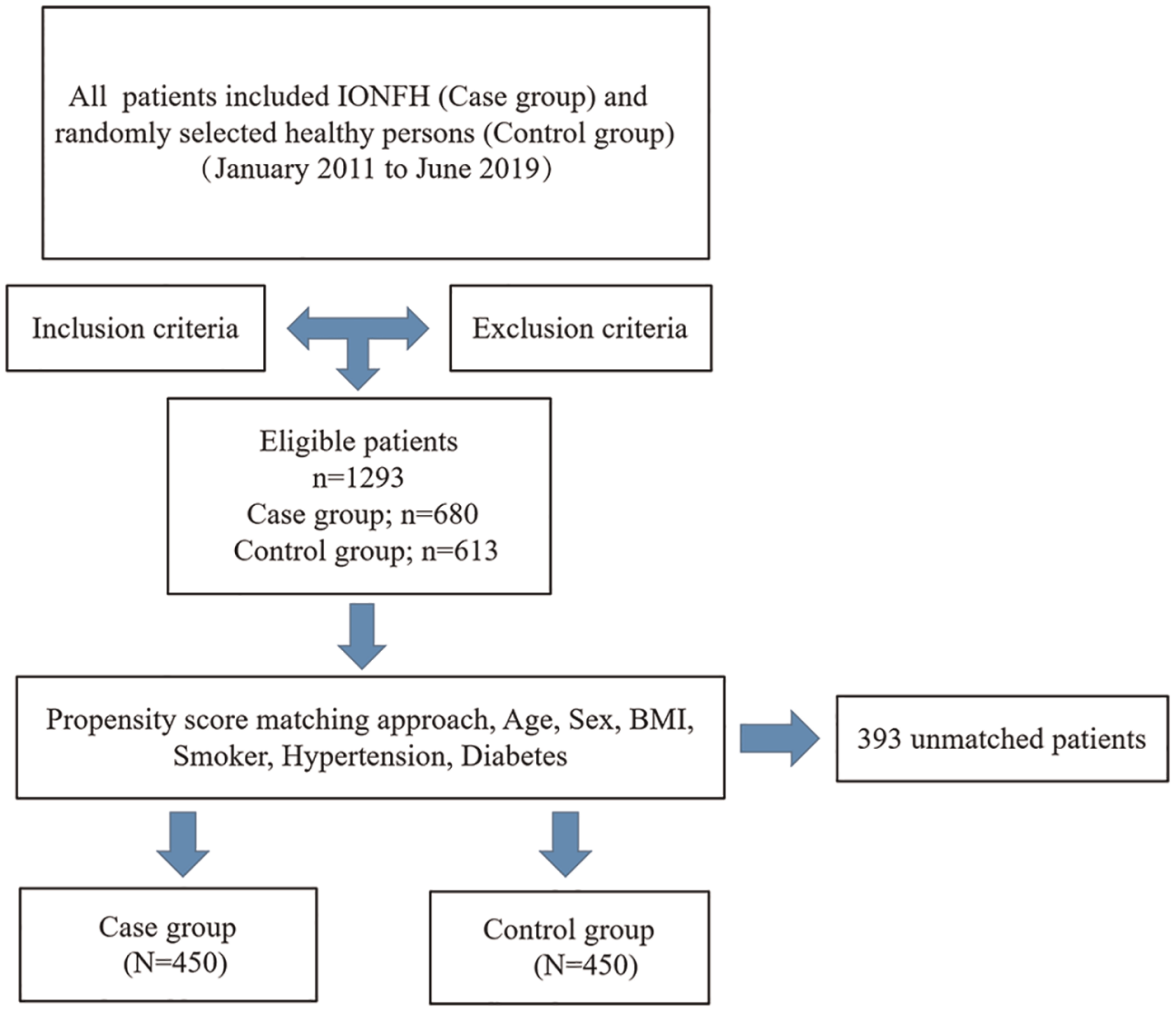
Patient selection flow chart. IONFH: idiopathic osteonecrosis of the femoral head.

Between January 2011 to June 2019, 1,293 patients who met the eligibility criteria were enrolled, of whom, 680 were diagnosed with IONFH (case group). In parallel, 613 healthy persons were selected by computerised randomisation from our institution's database who had been physically examined during the same period (control group). The inclusion criteria were as follows: (1) without any corticosteroid nor alcohol use and (2) with complete clinical medical records. The exclusion criteria were as follows: (1) with chronic kidney disease, chronic obstructive pulmonary disease, or cancer; (2) with incomplete or indeterminate clinical characteristics; (3) with corticosteroid-induced ONFH (the patient had a medication history of prednisolone or equivalent hormone >2 g for 3 months, with a steroid history of more than 3 months, and was diagnosed as ONFH within 2 years) and alcohol-induced ONFH (the average weekly drinking of pure alcohol >320 g, with a drinking history of more than 6 months, and diagnosed as ONFH within 1 year); (4) with hyperlipidaemia or taking lipid-lowering drugs and (5) taken heparin, oral anticoagulants, aspirin, or other platelet antiaggregants. To minimise possible confounding factors, propensity scores were matched at 1:1 with the calliper set at 0.2. The matched variables included the following demographic data: age, sex, body mass index (BMI), and a basic medical history of smoking, hypertension, and diabetes. After matching, 450 patients with IONFH comprised the case group and 450 healthy patients comprised the control group. A clear and intuitive flow chart of the patient selection process is shown in Figure 1.

### Data collection

IONFH patients are diagnosed at our institution in the outpatient clinic by an experienced clinician with a comprehensive analysis of the patient's clinical symptoms, physical signs and imaging ancillary examinations. After the diagnosis was confirmed, blood lipids and coagulation indicators were examined in the ward the next morning after admission. The following data were obtained retrospectively from medical records: age, sex, body mass index (BMI), diagnosis, smoking history, hypertension, and diabetes. Furthermore, data on total cholesterol (TC), triglyceride (TG), low-density lipoprotein (LDL), high-density lipoprotein (HDL), apolipoprotein A (apo-AI), apolipoprotein B (apo-B), activated partial thromboplastin time (APTT), prothrombin time (PT), fibrinogen (FIB) levels, and thrombin time (TT) were collected. An automatic biochemical analyser (OLYMPUS 5421, Olym-pus Corporation, Tokyo, Japan and Hitachi-7600, Hitachi Corporation, Tokyo, Japan) was used.

### Statistical analysis

SPSS (version 26.0 SPSS, Chicago, IL, USA) was used for propensity score matching and subsequent statistical analysis. Continuous variables are shown as mean ± standard deviation, and group variables are shown as numbers and percentages. Continuous variables were analysed using the two-tailed Student's independent t-test, and the Pearson chi-square test was applied to compare the percentages for binary data*. p* < 0.05 was considered as statistically significant.

## Results

### Propensity score matching results

Data of patients before and after the propensity score matching are presented in Tables 1, 2, respectively. The average age of patients was 61.5 ± 15.9 years in the initial cohort, 58.7 ± 12.7 years in the case group, and 64.5 ± 18.3 years in the control group. The average BMI in the initial cohort was 24.0 ± 2.0 and that in the case and control group was 23.6 ± 1.8 and 24.6 ± 2.1, respectively. In addition, a majority of patients in the control group were female (61.7%), whereas the gender distribution of females (46.2%) in the case group was lower. Before matching, except for smokers, we found statistical differences in other parameters. However, after matching the patients, both groups of 450 patients were created, and we found no significant difference in any of the parameters considered.

**Table 1:** Baseline characteristics of 1,293 patients in the case group and control group.

Variables	Total	Case gruop	Control group	*p* value
Number of patients	1293	680 (52.6%)	613 (47.4%)	
Age (years)	61.5 ± 15.9	58.7 ± 12.7	64.5 ± 18.3	<0.001
BMI (kg/m^2^)	24.1 ± 2.0	23.6 ± 1.8	24.6 ± 2.1	<0.001
Female (%)	692 (53.5%)	314 (46.2%)	378 (61.7%)	<0.001
Smoking (%)	162 (12.5%)	95 (14.0%)	67 (10.9%)	0.099
Hypertension (%)	308 (23.8%)	136 (20%)	172 (28.1%)	0.001
Diabetes (%)	120 (9.3%)	41 (6%)	79 (12.9%)	<0.001

**Table 2:** Baseline characteristics of case group and control group by 1:1 matching on propensity score.

Variables	Total	Case gruop	Control group	*p* value
Number of patients	900	450 (50%)	450 (50%)	
Age (years)	60.9 ± 15.4	60.3 ± 13.4	61.6 ± 17.1	0.215
BMI (kg/m^2^)	24.0 ± 2.0	23.9 ± 1.7	24.1 ± 2.3	0.138
Female (%)	513 (57%)	256 (56.9%)	257 (57.1%)	0.946
Smoking (%)	91 (10.1%)	49 (10.9%)	42 (9.3%)	0.439
Hypertension (%)	205 (22.8%)	105 (23.3%)	100 (22.2%)	0.691
Diabetes (%)	69 (7.7%)	39 (8.7%)	30 (6.7%)	0.260

### Comparison of blood lipid levels between the two groups

Based on propensity score matching of the whole cohort, we identified blood lipid levels associated with IONFH. As shown in Table 3, levels of TC, TG, LDL, apo-B, and the LDL/HDL ratio in the case group were 4.39 ± 0.87 mmol/L, 1.22 ± 0.59 mmol/L, 2.67 ± 0.75 mmol/L, 0.91 ± 0.23 g/L and 2.27 ± 0.88, respectively, which were significantly higher than those in the control group. Conversely, the HDL and apo-AI levels were 1.25 ± 0.34 mmol/L and 1.17 ± 0.24 g/L, respectively, which were significantly lower than those in the control group. All differences in blood lipid levels were statistically significant.

**Table 3:** Comparison of serum lipid indicators between groups.

Variables	Case gruop	Control group	*p* value
Number of patients	450	450	
TC (mmol/L)	4.39 ± 0.87	4.21 ± 0.87	0.002
TG (mmol/L)	1.22 ± 0.59	0.91 ± 0.37	<0.001
LDL (mmol/L)	2.67 ± 0.75	2.43 ± 0.71	<0.001
HDL (mmol/L)	1.25 ± 0.34	1.34 ± 0.35	<0.001
LDL/HDL	2.27 ± 0.88	1.93 ± 0.76	<0.001
Apo-AI (g/L)	1.17 ± 0.24	1.43 ± 0.30	<0.001
Apo-B (g/L)	0.91 ± 0.23	0.78 ± 0.22	<0.001

TC, total cholesterol; TG, triglyceride; LDL, low density lipoprotein; HDL, high density lipoprotein; Apo-AI, apolipoprotein A; Apo-B, apolipoprotein B.

### Comparison of coagulation levels between the two groups

Given that the pathogenesis of IONFH is associated with interruption of blood supply by the hypercoagulable state, we analysed the effect of four blood coagulation factors on IONFH. The APTT and PT levels in the case group were lower than those in the control group, though the differences were statistically insignificant (Table 4). In addition, the FIB levels and TT of the case group were higher than those of the control group. However, only the difference in FIB levels of both groups was significant, while the difference in TT was insignificant.

**Table 4:** Comparison of four coagulation indicators between groups.

Variables	Case gruop	Control group	*p* value
Number of patients	450	450	
APTT (s)	26.89 ± 4.68	27.41 ± 4.07	0.076
PT (s)	10.82 ± 1.64	11.03 ± 1.96	0.082
FIB (g/L)	2.97 ± 0.78	2.53 ± 0.72	<0.001
TT (s)	20.17 ± 2.38	19.93 ± 2.13	0.111

APTT, activated partial thromboplastin time; PT, prothrombin time; FIB, fibrinogen; TT, thrombin time.

## Discussion

This study was based on propensity score matching to compare differences in blood lipid levels and coagulation factors between patients with IONFH (case group) and healthy adults (control group). The results showed that TC, TG, LDL, Apo-B, and FIB levels and the LDL/HDL ratio in the case group were higher than those in the control group, whereas HDL and Apo-AI levels were significantly lower in the case group than in the control group. Our results emphasise upon the relationship between blood lipid and coagulation function in patients with IONFH and provide an avenue for exploring the mechanism of IONFH. From our results, we ascertain that in these IONFH patients, disease progression would be effectively delayed and patients would be prevented from requiring total hip arthroplasty (19–21).

Although the pathophysiological mechanism of IONFH remains unclarified, previous studies have shown that imbalances in blood lipid metabolism are indispensably associated with ONFH (22–24). In an animal experimental model of ONFH (25), an increase in blood lipid levels was the first change observed, which supports the notion that lipid levels may be related to osteonecrosis occurrence. Furthermore, in a related study, the cholesterol-lowering drug lovastatin could prevent the development of osteonecrosis in chickens *in vivo* by inhibiting the effects on chicken fat-specific gene expression (26). This is consistent with our findings that an increase in TC levels in peripheral blood is associated with an increased risk of IONFH. Some studies concluded that TG and LDL are independent risk factors and diagnostic criteria for ONFH (27, 28). Interestingly, our data are also consistent with previously published results described above; that is, TG and LDL levels in the case group were significantly higher than those in the control group. Additionally, Miyanishi et al. (29) reported that in a rabbit osteonecrosis model, the LDL/HDL cholesterol ratios of the osteonecrosis group were higher. Our novel findings are noteworthy as they support the hypothesis that higher LDL/HDL cholesterol ratios may also contribute to the pathogenesis of IONFH. On the other hand, apolipoproteins bind to lipids, they are present in plasma as lipoproteins, and mediate lipid transport through interactions with cellular receptors (30). Particularly, Apo-AI and Apo-B are two key proteins of lipid metabolism involved in ONFH (14), but their role in IONFH remains unclear. Our results suggest that both decreased Apo-AI and elevated Apo-B are risk factors for IONFH, which supports Hao's apolipoprotein gene polymorphism report that has a susceptibility to IONFH (31). Taken together, IONFH was highly associated with blood lipid metabolism.

There is considerable evidence to conclude that coagulation function affects the pathogenesis of ONFH (15, 16). In this study, the levels of coagulation factors affecting IONFH were evaluated and the results showed that only FIB levels were significantly higher in the IONFH population than in the control group (*p* < 0.05), while other indicators such as APTT, PT, TT were statistically insignificant (*p* > 0.05), which is consistent with the results of previous studies that described the association between coagulation function and IONFH (16, 32). For example, Glueck et al. (13) stated that genetic propensity to thrombosis and fibrinolysis are risk factors for IONFH. Moreover, Gagala et al. (33) also highlighted that inherited hypofibrinolysis is a risk factor for IONFH in the Polish population. Since IONFH has a close association with the coagulation level of FIB, intravascular coagulation function might be affecting its occurrence and development, which needs further evaluation.

Regarding the pathogenesis of IONFH caused by lipid metabolism and coagulation function, the findings of this study are consistent with the theory that injury of vascular endothelial cells and fat embolism increase pressure on the femoral head, which ultimately reduces the blood supply. First, due to damage to the vascular endothelial cells by hyperlipidaemia and the formation of a prethrombotic state, the ability of vascular endothelial cells to synthesise nitric oxide is decreased, which disrupts vasoconstriction and vasodilation (34). Second, hypercholesterolaemia with elevated serum cholesterol levels, fatty deformities in the liver, and systemic fat embolism, may cause an increased intraosseous pressure that destroys the microcirculation in the femoral head (35, 36). Third, high triglyceride levels are a risk factor for ischaemic heart disease and stroke (37, 38), which can partly or totally block the blood flow to the femoral head, while patients with IONFH tend to develop asymptomatic IONFH through similar mechanisms (28). Finally, the commonality in IONFH cases is the compromised blood flow to the affected area. All the above mechanisms can ultimately result in reduced blood flow to the femoral head, which can lead to bone ischaemia and death (9, 39, 40).

Corticosteroid-induced ONFH, alcohol-induced ONFH, and IONFH are the three main etiologies of nontraumatic ONFH. Corticosteroid- and alcohol-induced ONFH with lipid metabolism and coagulation have been reported extensively in the previous literature. However, only a few reports have explored the relationship between lipid metabolism and coagulation with IONFH. In this study, we performed statistical analysis based on a large sample of data collected at our institution and demonstrated that IONFH is closely associated with lipid metabolism and coagulation function. It provides a channnel to be able to explore the mechanism of action of nontraumatic ONFH as a whole in the future, and also provides potential ideas for the prevention of ONFH.

Despite these findings, this study has some limitations. First, this study was a single-centre retrospective case-control trial, not a randomised controlled trial. Although the sample size is relatively large, it had some limitations. Second, although propensity score matching was used to control certain confounding factors, uncontrolled selection and recall bias was possible; therefore, a prospective randomised study would better analyse the relationship of IONFH with blood lipid metabolism and coagulation function.

## Conclusion

From our results, we suggest that the occurrence of IONFH is highly associated with blood lipid levels and coagulation factors; thus, it provides an avenue of thought for exploring the mechanism of IONFH. Moreover, the specific effects of blood lipid metabolism and coagulation function on IONFH need further analysis.

## Data Availability

The original contributions presented in the study are included in the article/**Supplementary Material**, further inquiries can be directed to the corresponding author/s.
